# Angelica Essential Oil Loaded Electrospun Gelatin Nanofibers for Active Food Packaging Application

**DOI:** 10.3390/polym12020299

**Published:** 2020-02-02

**Authors:** Ying Zhou, Xiaomin Miao, Xingzi Lan, Junren Luo, Tingting Luo, Zhixin Zhong, Xifeng Gao, Zihui Mafang, Junjie Ji, Han Wang, Yadong Tang

**Affiliations:** 1 School of Biomedical and Pharmaceutical Sciences, Guangdong University of Technology, Guangzhou 510006, China; 2 Guangdong Provincial Key Laboratory of Micro-nano Manufacturing Technology and Equipment, School of Electromechanical Engineering, Guangdong University of Technology, Guangzhou 510006, China; 3 School of Biotechnology and Health Sciences, Wuyi University, Jiangmen 529020, China

**Keywords:** gelatin, angelica, electrospinning, nanofiber, hydrophobicity, bioactivity, food packaging

## Abstract

The development of food packaging possessing bioactivities which could extend the shelf life of food has gained increased interest in recent years. In this study, gelatin nanofibers with encapsulated angelica essential oil (AEO) were fabricated via electrospinning. The morphology of gelatin/AEO nanofibers was examined by scanning electron microscopy (SEM) and the addition of AEO resulted in the increase of fiber diameter. The proton nuclear magnetic resonance (^1^H-NMR) spectra were measured to confirm the presence of AEO in nanofibers. The hydrophobic property of gelatin nanofibers was also found to be improved with the addition of AEO. The nanofibers incorporated with AEO showed significant antioxidant activity and inhibitory effect against both Gram-negative and Gram-positive bacteria in a concentration dependent manner. Furthermore, the 3-(4,5-dimethyl-2-thiazolyl)-2,5-diphenyl-2-H-tetrazolium bromide (MTT) assay demonstrated that the developed gelatin/AEO nanofibers revealed no cytotoxicity effect. Thus, gelatin nanofibers incorporated with AEO can be used as potential food packaging.

## 1. Introduction

Nowadays, petrochemical-based plastics are widely used as food packaging, however they are not environmentally-friendly, recyclable and biodegradable, resulted in serious environmental problems [1,2,3]. Biodegradable materials derived from natural plants and animals are more and more studied and potentially widely applicable to food packaging [4,5,6].

Gelatin is one such material. It is a natural Generally Recognized as Safe (GRAS) polymer, which can be extracted from collagen, one of the most abundant protein in animals, and has been widely used in food, pharmaceutical, cosmetic industries [7,8]. However, as food packaging, the hydrophilic nature of gelatin limits its application due to its poor moisture barrier property [9]. Besides, gelatin does not possess enough bioactivities for food preservation. In recent years, biodegradable materials incorporated with active substances, such as antioxidant and antimicrobial agents are drawing increasing attention in the field of food packaging [10,11,12,13]. The active package allows to better protect food from the external environment, and extend the food shelf-life and safety [14].

Essential oils (EOs), which are extracted from aromatic plants, have been widely studied in the food and pharmaceutical fields not only because they are natural GRAS products but also due to their various bioactive properties such as antioxidant, antimicrobial, anti-tumor, and anti-inflammatory [15,16,17]. Several studies have reported the incorporation of EOs in food packaging, which showed the efficiency against microorganisms and oxidants and the benefits on food preservation [18,19,20]. The essential oil of *Angelica sinensis* (Oliv.) herb, known as Dong-gui in Chinese, has been demonstrated to possess outstanding antibacterial and antioxidant activities [21,22], which should be effective as a potential bioactive additive in food packaging. However as far as we know, the application of Angelica essential oil (AEO) in food packaging has not been reported yet.

In our previous study, the gelatin nanofibers encapsulated with peppermint and chamomile essential oil were successfully fabricated, which showed great potential as active food packaging [23]. In this study, novel AEO-loaded gelatin nanofibers were prepared still using electrospinning technique to avoid the rapid evaporation loss of volatile AEO in conventional gelatin casting process, as well as to enhance the bioactivities resulted from the nanostructure. The electrospinning solution properties after AEO was added, including the conductivity and viscosity, were firstly studied. The morphology, chemical structure and hydrophilicity of AEO-loaded nanofibers were characterized by scanning electron microscopy (SEM), proton nuclear magnetic resonance (^1^H-NMR) and water contact angle (WCA) measurements, respectively. The antioxidant and antibacterial activities were evaluated by 2,2-diphenyl-1-picrylhydrazyl hydrate (DPPH) radical scavenging assay and the dynamic contact method against *Escherichia coli* (*E. coli*) and *Staphylococcus aureus* (*S. aureus*), respectively. Finally, the cytotoxicity of the AEO/gelatin nanofiber was investigated by MTT assay.

## 2. Materials and Methods

### 2.1. Materials

Gelatin powder (*M*_w_ ≈ 60 kDa, G2625, Sigma-Aldrich, Shanghai China), 2,2-Diphenyl-1-picrylhydrazyl (DPPH, *M*_w_ = 394.32, 97% purity, D9132) were purchased from Sigma-Aldrich (Shanghai, China). Angelica essential oil (AEO, 98% purity) was from Jiangxi Cedar Natural Medicinal Oil Co., Ltd., Ji’an, China. Glacial acetic acid (*M*_w_ = 60.05, 99% purity) was provided from Tianjin Jindong Tianzheng Fine Chemical Reagent Factory, Tianjin, China. Phosphate buffer saline (PBS), dulbecco’s modified eagle medium (DMEM), fetal bovine serum (FBS), penicillin/streptomycin (P/S) and trypsin were obtained from Thermofisher Scientific (Guangzhou, China). (4,5-cimethylthiazol-2-yl)-2, 5-diphenyl tetrazolium bromide (MTT, *M*_w_ = 414.32, 98% purity) was from Shanghai Yuanju Biotechnology Co., Ltd, Shanghai, China. Muller Hinton (MH) broth and agar nutrient broth were supplied from Guangdong Huankai Microbiology Technology Co., Ltd., Guangzhou, China.

### 2.2. Preparation of Electrospun Nanofibers

**Figure 1:**
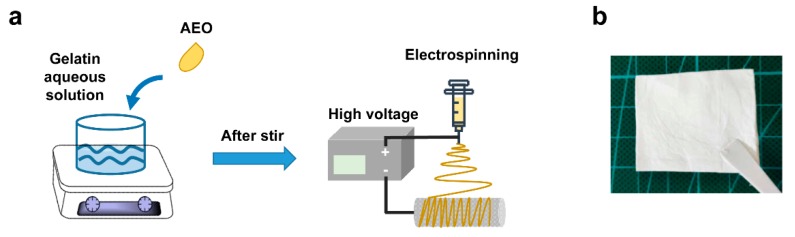
(**a**) Schematic of Angelica essential oil (AEO)/gelatin nanofiber fabrication by electrospinning; (**b**) Photograph of a piece of AEO/gelatin nanofibrous membrane.

The schematic of electrospun nanofiber preparation is shown in Figure 1a. Electrospinning solutions were prepared by firstly dissolving 12% *w/v* gelatin in 88% *v/v* acetic acid aqueous solution, and then added with 0%, 3%, 6% or 9% *v/v* of AEO. The conductivity of gelatin-AEO solution was measured with a conductivity meter (DDS-11A, Shanghai Leici Instrument Company, Shanghai, China) at 25 °C by immersing the probe into gelatin-AEO solutions. The viscosity of the solutions was measured through a digital display viscosimeter (NDJ-5S, Shanghai Hengping Instrument Factory, Shanghai, China) at 25 °C using spindle L-3 at constant speed of 60 rpm. All measurements were performed in triplicates, and all data were statistically analyzed to be expressed as mean ± standard deviation (SD).

The electrospun solution was loaded into a 1 mL syringe and ejected at a pumping speed of 0.3 mL/h. A bias voltage of 15 kV was applied between the stainless steel needle (23-gauge) of the syringe and the collector at a distance of 20 cm. After electrospinning, the sample was dried overnight in a desiccator to remove the residual solvent and set aside for experiments.

### 2.3. SEM Characterization

The morphology of gelatin/AEO nanofibers was directly observed under scanning electron microscope (TM3030, Hitachi, Tokyo, Japan) without gold sputtering at an accelerating voltage of 15 kV. The diameter of nanofibers was measured by analyzing the SEM images with ImagePro Plus 6.0 soft imaging system. At least 150 fibers in each sample were randomly selected and measured for calculating the average diameter. Frequency distribution histogram and Gaussian fitting curve were made to quantify the fiber deposition accuracy. All data were shown as average diameter (AD) ± standard deviation (SD).

### 2.4. Chemical Characterization

To prove the existence of AEO in nanofibers, 20 mg of nanofiber was dissolved into 500 μL of DMSO-d6 and characterized with ^1^H NMR (DPX-400, Bruker) spectra at 400 MHz [24]. The chemical shifts (δ) were reported in parts per million (ppm) relative to residual DMSO-d6 (δ = 2.50, 1 H), and tetramethylsilane (TMS) was used as an internal standard. Mestrenova software (Mestrelab Research) was used to integrate the chemical shifts (δ) given in parts per million (ppm).

### 2.5. Water Contact Angle Measurement

The hydrophilicity of the firous mats was evaluated with an automatic contact angle meter (OCA100, Dataphysics, Filderstadt, Germany). Briefly, 2 μL of distilled water was dropped on the surface of nanofibrous mat and photographed immediately (*t* = 0). Each sample was tested at least three times at different locations for averaging.

### 2.6. Antioxidant Activity Study

The antioxidant activity of nanofibers was analyzed by testing the DPPH radical scavenging activity according to our previously reported method [23]. Briefly, nanofiber sample was immersed in 3 mL of DPPH solution (0.1 mmol/L in alcohol) for 30 min in darkness at room temperature. Then the absorbance of the solution was recorded with an UV-Vis spectroscopy (Lambda 25, PerkinElmer, Inc., MA, USA) at 517 nm. The DPPH scavenging activity was calculated as follows:

$$\mathrm{DPPH}\;\mathrm{scavenging}\;\mathrm{activity}\;\left(\%\right)=\frac{Control_{OD}-Sample_{OD}}{Control_{OD}}\times 100$$

### 2.7. Evaluation of the Antibacterial Activity of Gelatin/AEO Nanofibers

The antibacterial activity of the gelatin/AEO nanofibers was evaluated with a semi-quantitative shake flask method against two representative microorganisms, *E. coli* and *S. aureus* [25]. *E. coli* and *S. aureus* were cultured in Muller Hinton broth at 37 °C with gentle shaking (100 rpm), and after 24 h fresh colonies were used to prepare bacterial suspensions. The turbidity of bacterial suspension was adjusted to 0.5 McFarland standard (around 1.5 × 10^8^ CFU /mL). Then, 20 mg of each nanofibrous sample was cut and sterilized with UV irradiation for 20 min on each side, and soaked in 3 mL of fresh Muller Hinton broth containing 10 μL of the prepared bacterial suspension (5 × 10^5^ CFU/mL) at 37 °C for 24 h. The bacterial solution without any nanofiber was applied as a control. Finally, the growth of bacterial was indirectly measured by optical density at 625 nm with a UV-vis spectrometer (Lambda25, PerkinElmer, Inc., MA, USA). Then the antibacterial activity of nanofiber can be calculated as follows:

$$\mathrm{Antibacterial}\;\mathrm{activity}\;\left(\%\right)=\left(\frac{A_{c}-A}{A_{c}}\right)\times 100$$

where *A*_c_ is the absorbance of the bacterial solution in control group, while *A* is the absorbance of the bacterial solution with according nanofiber sample.

### 2.8. Biocompatibility Study of Gelatin/AEO Nanofibers

The biocompatibility of gelatin/AEO nanofibers was measured according to our previous reported method by MTT assay [26]. NIH/3T3 cells were cultured in DMEM with 10% fetal bovine serum (FBS), 100 U/mL penicillin and streptomycin at 37 °C with 5% CO_2_ in a humidified atmosphere incubator. The glass slide (22 × 22 mm) covered with gelatin/AEO nanofibers was sterilized by UV light for 15 min and placed in 6-well plate. Then 10^5^ NIH/3T3 cells were seeded on each nanofiber sample. After culture for 24 h and 48 h, the culture medium in each well was replaced by 2 mL of MTT solution (0.5 mg/mL). After incubation for 4 h, 2 mL DMSO was added to exchange the MTT solution in each well. Then the purple solution in each well was transferred into a 96-well plate and the absorbance was measured using a microplate reader at 570 nm (Multiskan, Thermo-Fisher, Waltham, MA, USA). Experiments were carried out in triplicate.

### 2.9. Statistical Analysis

Experiments were performed in triplicate (n = 3) and results were expressed as means ±SD. Statistical analyses were performed using GraphPad Prism 7. To determine the statistical difference, one-way analysis of variance (ANOVA) and Tukey’s post hoc test (*p* < 0.05) were employed.

## 3. Results and Discussion

### 3.1. Morphology of Gelatin/AEO Nanofibers

**Figure 2:**
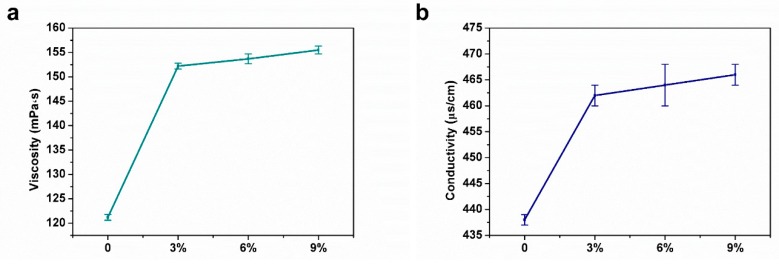
Viscosity (**a**) and conductivity (**b**) of the electrospinning solutions with varying AEO content (0%, 3%, 6%, 9% *v/v*). Results are mean ±SD (n = 3).

The gelatin nanofibers with encapsulated different content of AEO were prepared by electrospinning (Figure 1). Figure 1b shows the photograph of a piece of gelatin/AEO nanofibrous mat. Since the rheology and conductivity properties of electrospinning solution largely affect the stability of electrospinning process and the morphology of electropun nanofibers [27], the parameters of electrospinning solutions were firstly measured in our study. As shown in Figure 2, both the viscosity and conductivity of the electrospinning solution were enhanced with the addition of AEO, which resulted from the increased total ion amounts [28]. In general, the increase of viscosity resulted in less streching, thus larger fiber diameter, while the increase of conductivity led to improved streching, thus reduced fiber diameter [29].

**Figure 3:**
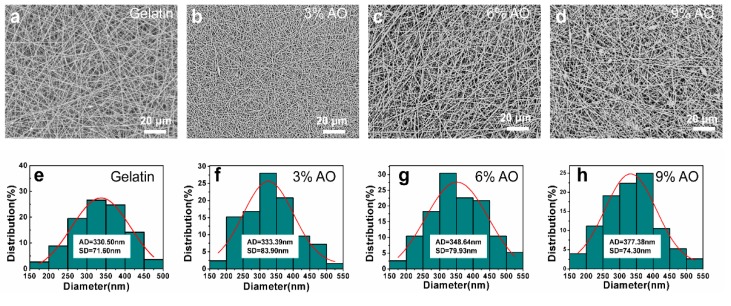
Representative SEM images (**a**–**d**) and fiber diameter distribution (**e**–**h**) of gelatin/AEO nanofibers with AEO ratio at 0%, 3%, 6% and 9% (*v/v*), respectively. Scale bar: 20 µm. Results are mean ±SD (n > 100).

During the experiment, the gelatin solutions containing 0% to 9% *v/v* AEO had good spinnability and resulted in uniform nanofibers, as shown in the SEM images in Figure 3a–d. After measurement, the diameter of nanofibers was found to be slightly enlarged from 330.50 ± 71.60 nm to 377.38 ± 74.30 nm with the increase of AEO content from 0% to 9% *v/v* (Figure 3e–h), which could be related to the change of electrospinning solution properties of viscosity and conductivity [27]. In general, the diameter of the nanofibers increases as the viscosity of the spinning solution increases, but decreases as the conductivity increases [30]. In our study, both the viscosity and conductivity of the spinning solution were enhanced with the increase of AEO content, which codetermined the final increased diameter of the nanofibers.

### 3.2. Chemical Characterization of Gelatin/AEO Nanofibers

**Figure 4:**
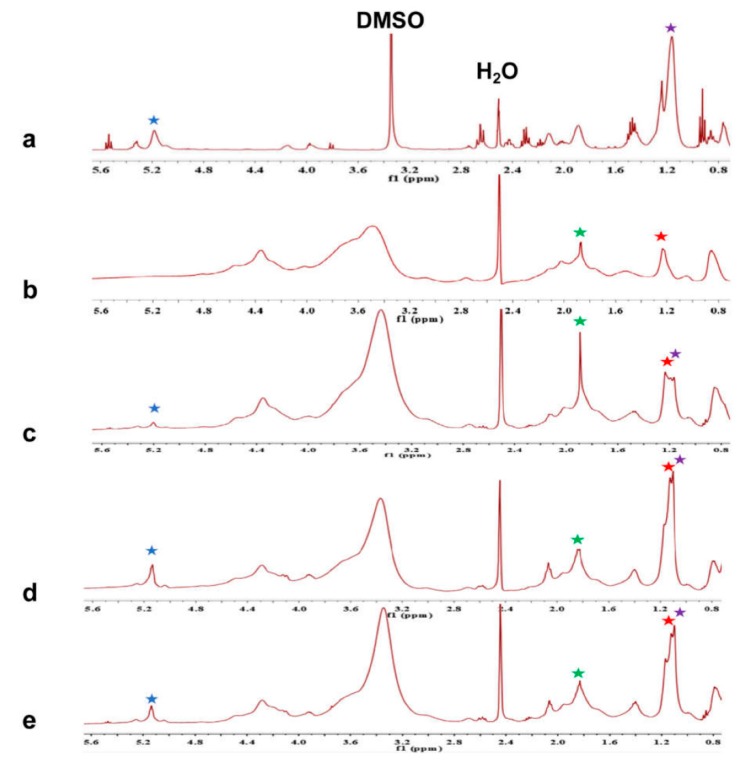
^1^H-NMR spectra of pure AEO (**a**) and gelatin/AEO nanofibers (**b**–**e**) with AEO ratio at 0%, 3%, 6%, 9% (*v/v*), respectively. (Protons used to prove the existence of gelatin and AEO are shown by star signs: green and red for gelatin; purple and blue for AEO).

To verify the presence of AEO in nanofibers after electrospinning, the chemical structure of AEO, gelatin, and gelatin/AEO nanofibers was characterized by ^1^H-NMR, as shown in Figure 4. It could be observed from Figure 4a that AEO has the characteristic peaks at 0.9–1.2 ppm and 5.1–5.4 ppm, corresponding to ―CH_3_ in E-3-butylidenephthalide, and =CH in ligustilide, respectively [31]. The evident peaks of gelatin spectrum in Figure 4b are at around 1.22 ppm and 1.93 ppm shifts, which belong to the ―CH_3_ of threonine and proline, respectively [32]. The spectra of gelatin/AEO nanofibers with 3%, 6% and 9% AEO are shown in Figure 4c–e, and all the characteristic peaks of gelatin and AEO could be found in the spectra of gelatin/AEO nanofibers. Besides, as the concentration of AEO increased, the characteristic peaks of AEO became much higher accordingly. The results demonstrated that AEO was successfully encapsulated in gelatin nanofibers.

### 3.3. Water Contact Angle Measurement

**Figure 5:**
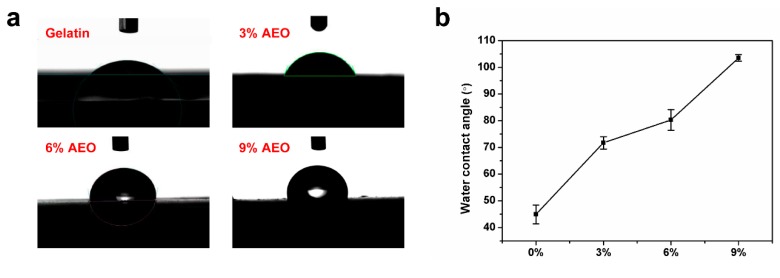
(**a**) Photographs of the water droplets on gelatin/AEO nanofibers in contact angle measurement (*t* = 0); (**b**) Contact angle values of the gelatin/AEO nanofibers. Results are mean ±SD (n = 3).

Water contact angle test was applied to investigate the surface wettability level of the gelatin/AEO nanofibrous mats (Figure 5). Due to the hydrophilic nature, electrospun gelatin nanofibers revealed low water contact angle (44.9 ± 3.48°), which largely limited its applications as food packaging because of the worse barrier property against moisture. With the addition of AEO, the water contact angle of nanofibers increased significantly, suggested the better hydrophobicity of nanofibers was achieved. Remarkably, the gelatin nanofibers containing 9% *v/v* AEO showed a water contact angle of 101.3 ± 5.55°, which is typically hydrophobic performance. It could be related to the hydrophobic property of essential oil and also further demonstrated the encapsulation of AEO in nanofibers [33]. Besides, the improved hydrophobicity suggested the better barrier property of gelatin/AEO nanofibrous membranes as packaging materials against moisture penetration.

### 3.4. Antioxidant Activity

**Figure 6:**
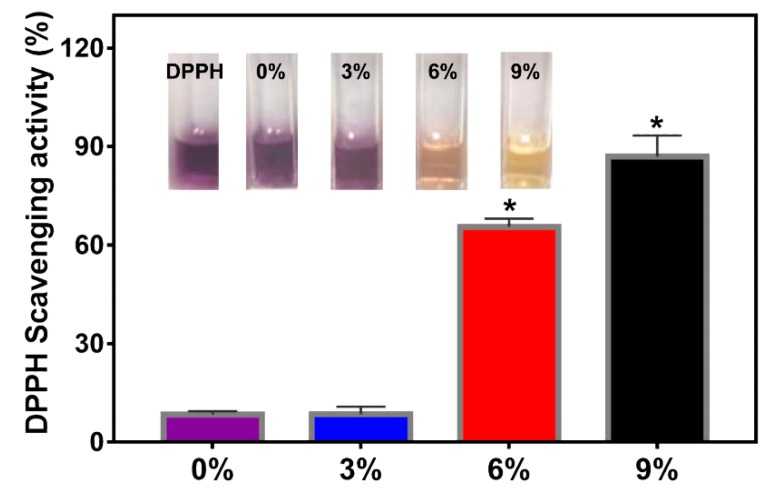
Antioxidant activity of the gelatin/AEO nanofibers and the photographs of the resulted DPPH solution after reaction with gelatin nanofibers with 0%, 3%, 6% and 9% *v/v* AEO. * *p* < 0.05 versus the control group. Results are mean ± SD (n = 3).

Antioxidants can serve as effective preservative in food-packaging applications. AEO has been proved to possess excellent antioxidant activity due mainly to the presence of E-3-butylidenephthalide [34]. In order to evaluate the antioxidant activity of gelatin/AEO nanofibers, DPPH radical scavenging assay was performed. As shown in Figure 6, compared with gelatin nanofiber group, nanofibers contained 3% *v/v* AEO displayed weak antioxidant activity. As the concentration of AEO increased, the antioxidant activity was enhanced drastically. Especially, the nanofiber membrane contained 9% *v/v* AEO exhibited the best radical scavenging activity (85% ± 6%). This result indicated that gelatin/AEO nanofibers possess outstanding antioxidant property and could protect food from oxidative decay.

### 3.5. Antibacterial Activity

**Figure 7:**
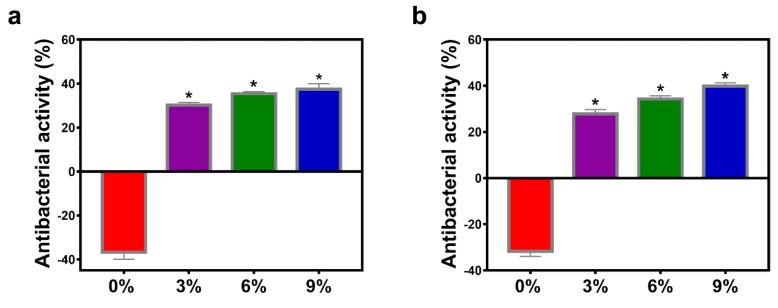
Antibacterial activity of the gelatin/AEO nanofibers with varying AEO content (0%, 3%, 6%, 9% (*v/v*)) against E. coli (**a**) and S. aureus (**b**), respectively. * *p* < 0.05 versus the control group. Results are mean ±SD (n = 3).

Microbial infestation is a major cause of food spoilage. It has been reported that AEO has effective and broad-spectrum antibacterial activity, which could be related to ligustilide, one of the main compounds of AEO [35]. The antibacterial activity of gelatin/AEO nanofibers tested by semi-quantitative shake flask method is presented in Figure 7. The gelatin nanofiber without AEO showed no antibacterial activity against *E. coli* and *S. aureus* after 24 h of incubation, and even facilitation to the growth of these two bacteria, which is consistent with the previous report [36]. In contrast, all the nanofibers containing AEO exhibited obvious AEO concentration-dependent inhibition effect on the growth of both *E. coli* and *S. aureus*. The result demonstrates that gelatin/AEO nanofiber membrane could effectively control the development of decay caused by microorganisms and is a promising candidate of active packaging for extending shelf life and maintaining food safety.

### 3.6. Biocompatibility Evaluation

**Figure 8:**
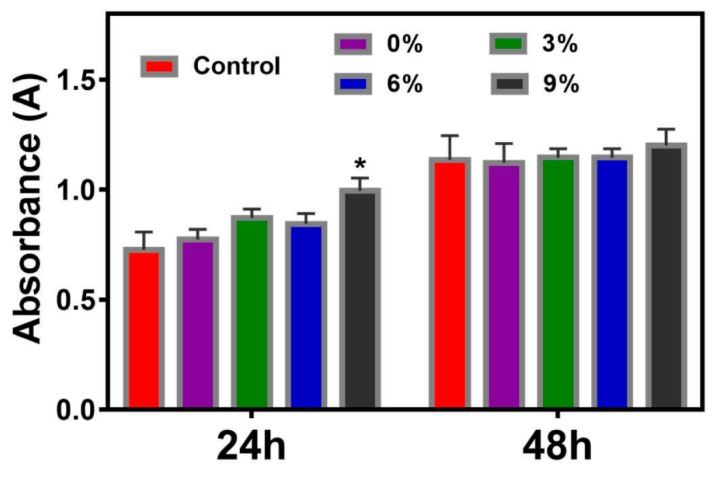
Viability of NIH-3T3 cells after cultured on gelatin/AEO nanofibers for 24 h and 48 h, respectively. * *p* < 0.05 versus the control group. Results are mean ±SD (n = 3).

To further verify the safety of gelatin/AEO nanofibers as active food packaging, the biocompatibility of the nanofibers was examined by MTT assay. As shown in Figure 8, after 24 h of culture, cells on nanofibers showed good and even better viability than that on glass slide. After culture for one more day, the OD values in all groups increased which suggested the proliferation of cells, and cells in nanofiber groups still exhibit better viability than the control. Besides, there was no obvious cell viability difference among nanofiber groups with different concentrations of AEO. These results suggest that the gelatin/AEO nanofibers have no cytotoxicity and are promising to be used as active food packaging.

## 4. Conclusions

In this study, we have fabricated gelatin/AEO nanofibers with a certain percentage of AEO (0%, 3%, 6%, 9% *v/v*) via electrospinning technique. SEM images revealed all the electrospun membranes possessed homogeneous nanofibrous morphology. ^1^H-NMR analysis confirmed the existence of AEO in the gelatin/AEO nanofibers. The addition of AEO reduced the wettability and enhanced the water barrier function of gelatin nanofibers. The radical scavenging assay and shake flask method indicated that the gelatin/AEO nanofibers have excellent antioxidant activity and good antibacterial function against both Gram-positive and Gram-negative bacteria. Moreover, MTT assay revealed that the non-cytotoxicity of gelatin/AEO nanofibers. These results indicate that the developed gelatin/AEO nanofibers show great potential as a food packaging material.
